# Action video games improve reading abilities and visual-to-auditory attentional shifting in English-speaking children with dyslexia

**DOI:** 10.1038/s41598-017-05826-8

**Published:** 2017-07-19

**Authors:** Sandro Franceschini, Piergiorgio Trevisan, Luca Ronconi, Sara Bertoni, Susan Colmar, Kit Double, Andrea Facoetti, Simone Gori

**Affiliations:** 1 Developmental and Cognitive Neuroscience Lab, Department of General Psychology, University of Padua, Padova, 35131 Italy; 2 Child Psychopathology Unit, Scientific Institute “E. Medea”, Bosisio Parini, Lecco, 23842 Italy; 3 Department of Languages and Literatures, Communication, Education and Society, University of Udine, Udine, 33100 Italy; 4 Center for Mind/Brain Sciences, University of Trento, Rovereto, Trento 38068 Italy; 5 Sydney School of Education and Social Work, University of Sydney, Sydney, NSW 2006 Australia; 6 Department of Human and Social Sciences, University of Bergamo, Bergamo, 24129 Italy

## Abstract

Dyslexia is characterized by difficulties in learning to read and there is some evidence that action video games (AVG), without any direct phonological or orthographic stimulation, improve reading efficiency in Italian children with dyslexia. However, the cognitive mechanism underlying this improvement and the extent to which the benefits of AVG training would generalize to deep English orthography, remain two critical questions. During reading acquisition, children have to integrate written letters with speech sounds, rapidly shifting their attention from visual to auditory modality. In our study, we tested reading skills and phonological working memory, visuo-spatial attention, auditory, visual and audio-visual stimuli localization, and cross-sensory attentional shifting in two matched groups of English-speaking children with dyslexia before and after they played AVG or non-action video games. The speed of words recognition and phonological decoding increased after playing AVG, but not non-action video games. Furthermore, focused visuo-spatial attention and visual-to-auditory attentional shifting also improved only after AVG training. This unconventional reading remediation program also increased phonological short-term memory and phoneme blending skills. Our report shows that an enhancement of visuo-spatial attention and phonological working memory, and an acceleration of visual-to-auditory attentional shifting can directly translate into better reading in English-speaking children with dyslexia.

## Introduction

Dyslexia is a specific impairment in the acquisition of reading and spelling abilities, despite normal intelligence and educational resources^1^. Although dyslexia prevalence is in part influenced by language rules and writing system characteristics, it is present both in deep and shallow alphabetic orthographies as well as in non-alphabetic languages^2^.

The most common explanation of dyslexia relates it to a specific disorder in auditory and phonological processing^1, 3^. During reading acquisition, speech-sound segmentation of spoken words and phonological working memory are both necessary to translate letter strings into a phonological code (i.e., phonological decoding). The semantic access of words cannot be obtained before the completion of these processes. Indeed, White *et al*.^4^ showed that phonological skills accounted for variation in literacy skill in children with dyslexia, and reviews of the relationship between phonemic awareness and word reading skills indicated that children with dyslexia show a deficit in phonological awareness in relation not only to typically developing children of the same age, but also to children matched in reading level (refs 5, 6, 7).

After a specific intervention targeting phonological decoding, many children with dyslexia can achieve functional reading skills, although reading speed is generally harder to remediate than accuracy deficit^8^. Extremely slow and serial phonological decoding has therefore been proposed as the core deficit in dyslexic readers across both shallow and deep orthographies^9^.

In addition to phonological deficits, difficulties in rapid orienting of the attentional spotlight are also considered core deficits in dyslexia, as was shown in different visual search and covert orienting tasks^10–16^. In alphabetic and non-alphabetic languages, the spotlight of visual attention in individuals with dyslexia is sluggish and weaker in comparison to chronological-age and reading level controls^17–22^. Attentional deficits in individuals with dyslexia have been found in spatial and in the temporal processing of sequence of stimuli, using backward masking and attentional blink tasks^15, 23–25^. During early development, these abilities predict future reading skills both in shallow^13, 14, 26^ and in deep orthographies^16^, confirming a causal link between early visuo-attentional deficits and future reading difficulties (refs 1, 25, 27).

Besides showing visual difficulties in serial processing of rapid stimuli, it has been demonstrated that children with dyslexia also have difficulties in serial processing of rapid auditory stimuli^28, 29^. Therefore, a sluggish domain-general attentional shifting is an alternative explanation to phonological decoding deficits^30–32^. This could also explain the typical deficits in perceptual noise exclusion found in visual^33–36^ and in auditory stimuli^37, 38^ both in children with dyslexia and in children with specific language impairment. Finally, it is crucial to focus on the role played by spatial and temporal attention in multisensory integration^39^ in order to better understand the complex developmental mechanisms involved in reading acquisition^30, 40^. Similarly to the cross- and multisensory mechanisms that integrate speech and lip movements during language development, the activation of a specific neurocognitive mechanism is at the basis of the integration of congruent letters and speech-sounds in reading acquisition^41, 42^. These cross- and multisensory integration mechanisms - strictly involved in reading acquisition - are able to change the phonological coding in language-specific cortical areas, such as the left planum temporale^43^.

Harrar *et al*.^44^ have recently demonstrated that English adults with dyslexia - compared with subjects without dyslexia - exhibit a deficit in multisensory integration and tend to distribute their attention asymmetrically between auditory and visual modalities. In particular, individuals with dyslexia present difficulties in attentional shifting from visual to auditory, but not from auditory to visual stimuli^44^.

Interestingly, several studies reported the beneficial effects of specific kinds of video games not only on spatial and temporal attention^45^, but also on reading abilities^46^. These video games are identified as action video games (AVG) and are distinguished from other types of video games (Non Action Video Games - NAVG) for specific characteristics like speed, high sensory-motor load, and presentation of multiple, peripheral, rapidly moving, spatio-temporally unpredictable stimuli^45^. In particular, by activating both spatial and temporal attention at the same time, AVG enable subjects to enlarge the size of their useful field of view^47^ and to improve the fast discrimination of a rapid sequence of visual stimuli^47^ as well as the perception of visual global motion in noise^26, 48, 49^. Compared to controls who were trained with NAVG, Italian children with dyslexia who played AVG showed an improvement in visuo-spatial and temporal attentional shifting matched with an improvement in reading speed without any increase in reading errors rate^20^. Furthermore, AVG training in Italian children with dyslexia improved global motion perception, word recognition and phonological decoding efficiency^26^, Experiment 3).

Bavelier, Green and Seidenberg^50^ argued that such reading improvements were to be attributed to the very high degree of spelling-sound consistency in Italian orthography and pointed out the unlikelihood of similar results in deep orthographies such as English, where letters are pronounced differently according to their combination with the other letters in the string. In addition, Bavelier *et al*.^50^ suggested that there could be a variety of underlying neurocognitive deficits responsible for mediating the efficacy of an AVG training for reading improvement in Italian children with dyslexia.

Although children and adults with dyslexia can also exhibit visual working memory deficits^51^, working memory is generally found to be impaired in the auditory-phonological domain^5, 6^. Given the proposed visual mechanisms underlying AVG effects, one would predict such training to be more efficient in dyslexic individuals with pronounced deficits in visual attention and perception, with little or no benefit in phonological processing and memory deficits. However, as demonstrated by Sperling *et al*.^33^, in languages with deep orthography, attentional deficits in dyslexia are expressed by a difficulty in extracting information from the surrounding perceptual noise. Moreover, AVG training could improve the domain-general learning mechanisms involved in perceptual noise exclusion both in the visual and auditory modalities, together with their multisensory integration^26, 45, 48, 52, 53^.

Therefore we predicted that, regardless of orthography, AVG training in children with dyslexia would improve visual, auditory and cross-modal attentional shifting, with cascading effects on audio-visual processing and phonological working memory, as well as reading speed.

In our study, we matched two groups of English-speaking children with dyslexia and tested their reading skills, phonological working memory, visuo-spatial attention, auditory, visual and audio-visual stimuli localization and cross-sensory attentional shifting before and after playing AVG or NAVG.

## Results

### Reading abilities

The execution time (in sec.) and the number of errors in the lists of words and pseudowords were analyzed to measure the effect of the two different trainings on reading abilities.

#### Word recognition

Word reading ability improvements were analyzed using a 2 (time: pre vs. post) X 2 (intervention group: AVG vs. NAVG) analysis of Covariance (ANCOVA). In the first ANCOVA, the dependent variable was execution time. To exclude the possible effects of educational experience and reading impairment severity, chronological age and reading performance (the mean between “sight word” and “phonemic awareness” TOWRE-2 z-scores) in T1 were controlled by entering them as covariates.

**Figure 1:**
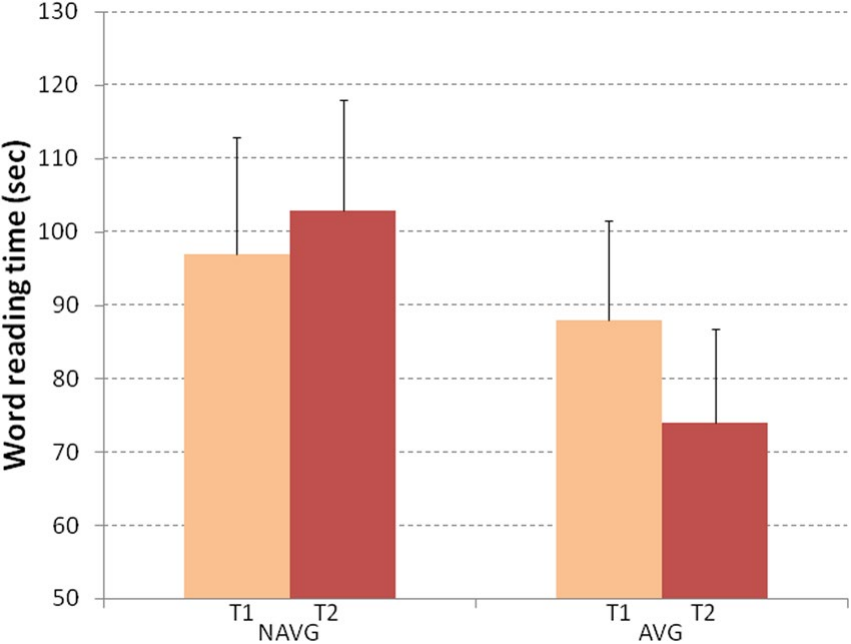
Word reading performance was measured before (T1) and after (T2) NAVG and AVG trainings in English-speaking children with dyslexia. The time for word recognition was significantly reduced only after AVG training. Error bars represent standard errors.

The time X group interaction was significant (F_(1,24)_ = 4.81, p = 0.038 η^2^ = 0.17; see Fig. 1). Post-hoc comparisons showed that participants in the AVG group significantly decreased their reading time (pre-training mean = 88, SE = 13; post-training mean = 74, SE = 13 p = 0.024, Cohen’s d, using the formula: mean in T1- mean in T2/pooled SD, was 0.27), whereas NAVG group did not show any significant differences between pre- (mean = 97, SE = 16) and post-training (mean = 103, SE = 15). We also calculated the Cohen’s d comparing the changes (T2-T1) of each group, using the formula for independent sample (mean of AVG group) − (mean of NAVG)/(pooled standard deviation) which resulted 0.86.

The same ANCOVA model was performed using number of errors as the dependent variable. No main effect or interaction was significant (Table 1).

**Table 1:** Mean (and Standard Deviation) of AVG and NAVG group performance in word and pseudoword reading tasks in time (sec.) and number of errors, both before (T1) and after (T2) the videogame trainings.

Group	Evaluation	Word reading		Group	Evaluation	Phonological decoding	
		Time	Errors			Time	Errors
		Mean (SD)	Mean (SD)			Mean (SD)	Mean (SD)
AVG	T1	88 (54)	13 (6)	AVG	T1	86 (35)	16 (6)
	T2	74 (51)	12 (7)		T2	69 (40)	16 (7)
NAVG	T1	97 (55)	10 (6)	NAVG	T1	79 (36)	16 (6)
	T2	103 (52)	10 (7)		T2	82 (41)	17 (6)

#### Phonological decoding

**Figure 2:**
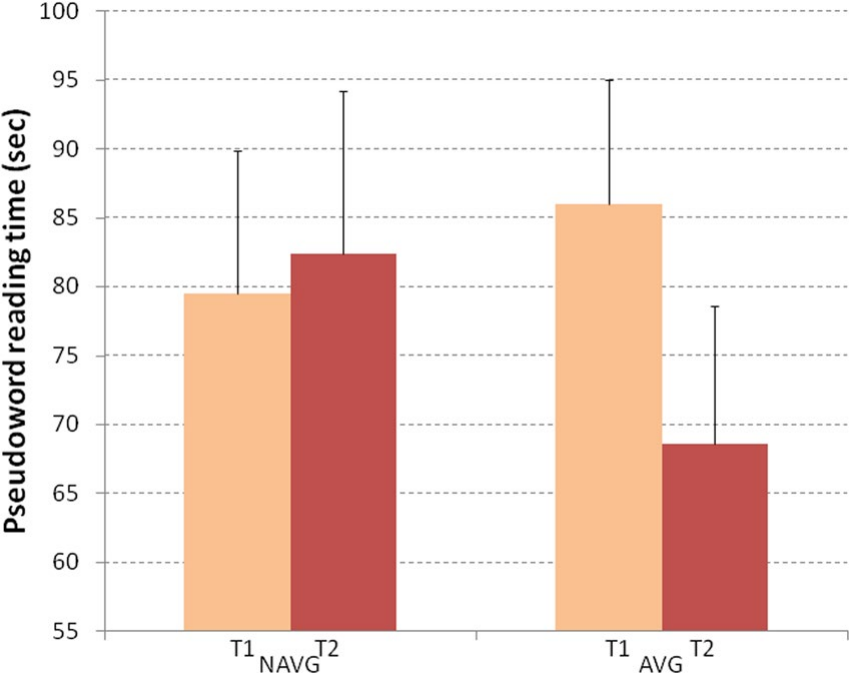
Pseudoword reading performance was measured before (T1) and after (T2) NAVG and AVG trainings in English-speaking children with dyslexia. The time for phonological decoding was significantly reduced only after AVG training. Error bars represent standard errors.

The ANCOVA model described above was applied to pseudoword reading abilities. Using reading time (in sec.) as the dependent variable. The time X group interaction was significant (F_(1,24)_ = 6.162, p = 0.02 η^2^ = 0.204; see Fig. 2). Post-hoc comparisons showed that children with dyslexia in the AVG group significantly decreased their phonological reading time (pre-training mean = 86, SE = 9; post-training mean = 69, SE = 10, p = 0.003, Cohen’s d = 0.45), whereas participants in the NAVG group did not show any significant difference between pre- (mean = 79, SE = 10) and post-training (mean = 82, SE = 12). Comparing the mean changes between AVG and NAVG groups, Cohen’s d was = 0.98.

We ran a similar ANCOVA, differing only in the dependent variable (number of errors). No main effect or interaction was significant (Table 1).

### Training efficacy on speed and errors in reading tasks: Individual results

**Figure 3:**
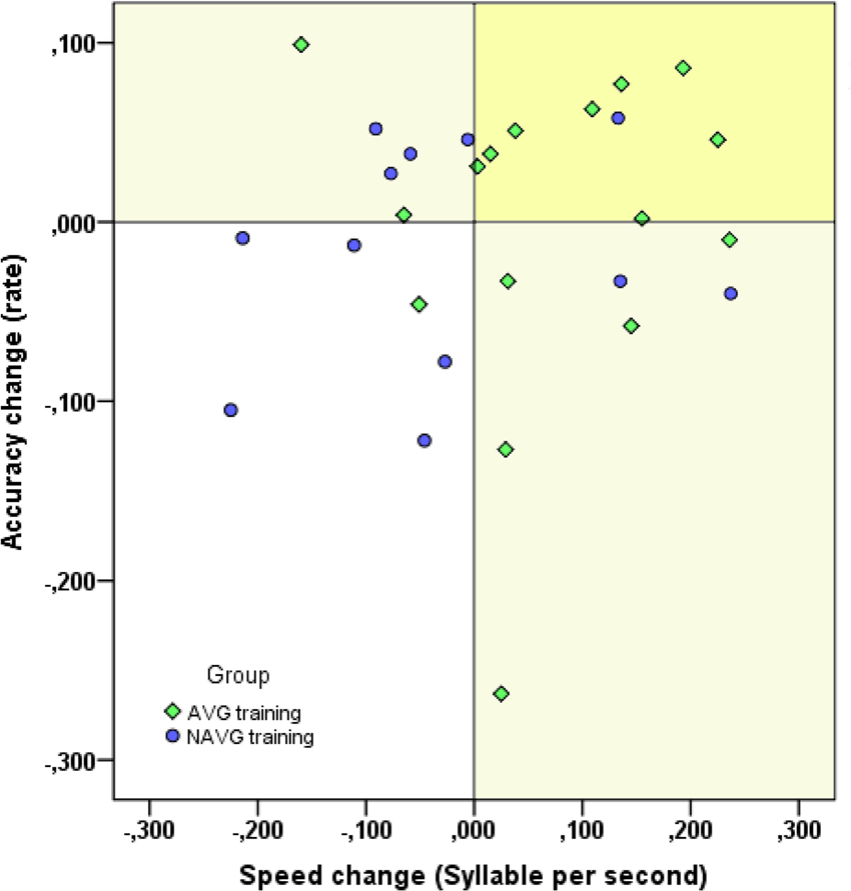
The mean of word and pseudoword speed (syllable per sec.) change and accuracy (rate) change between reading performance in T2 and T1 is reported for each child of the AVG (green diamonds) and NAVG (blue circles) groups. The yellow part contains participants that showed a positive direction in both speed and accuracy rate.

The aim of this analysis is to control the effect of the video game training on reading speed and accuracy for each participant. The mean of word and pseudoword change (in syllable per second) and accuracy (rate) between reading in T2 and T1 are reported in Fig. 3. Eighty-one percent of the AVG participants had an improvement in reading speed (Binomial test, p = 0.021) and 63% of them had an improvement in reading accuracy (Binomial test, p = 0.454) after training. Fifty percent of the AVG participants improved both in speed and in accuracy. Only one of the AVG participants had a worse performance in reading speed and accuracy. In contrast, only 25% of the NAVG participants had an improvement in speed (Binomial test, p = 0.146) and 42% of them had an improvement in accuracy (Binomial test, p = 0.774). Only 8% of the NAVG participants showed an improvement both in speed and accuracy. Forty-two percent of the NAVG participants showed a negative change of reading speed and accuracy.

The odds ratio is the ratio of the chance of an event occurring in one group (i.e., efficacy improvements in the AVG group) to the odds of it occurring in another group (i.e., efficacy improvements in the NAVG group). Odds ratio was 11 (95% confidence interval from 1.14 to 106.43), indicating the good efficacy of the AVG training in dyslexia.

### Auditory-phonological working memory

The number of correct items in the phonological short-term memory and phoneme blending task were analyzed to measure the effect of the two different trainings on auditory-phonological working memory.

Accuracy in auditory-phonological working memory was analyzed using a 2 (time: pre vs. post) X 2 (task: memory and blending) X 2 (intervention group: AVG vs. NAVG) ANCOVA. To exclude the possible effect of educational experience, chronological age was controlled by entering it as covariate.

**Figure 4:**
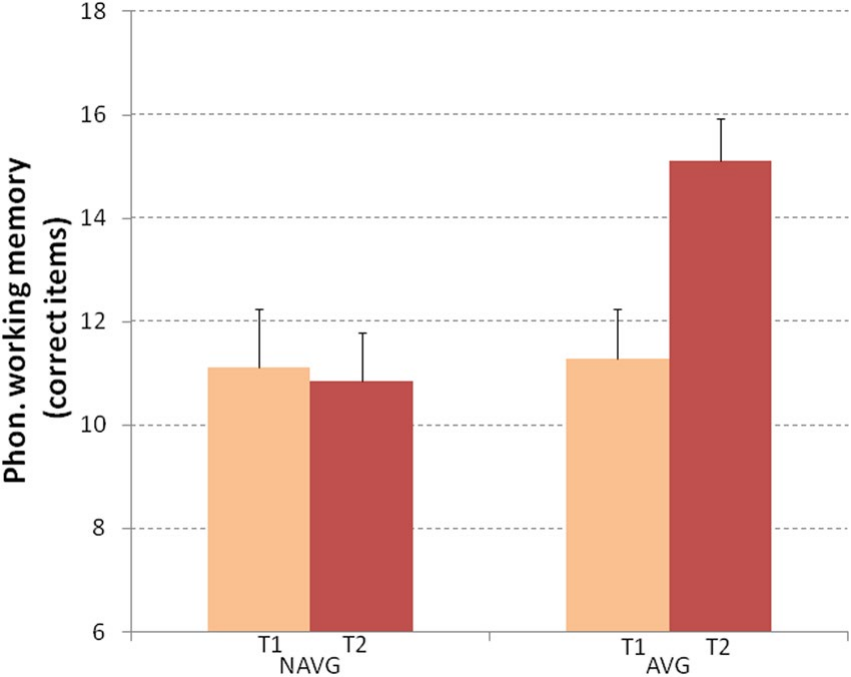
Auditory-phonological working memory (i.e., phonological short-term memory and phoneme blending) were measured before (T1) and after (T2) NAVG and AVG trainings in English-speaking children with dyslexia. Significant improvement in auditory-phonological working memory was observed only after AVG training. Error bars represent standard errors.

The time X group interaction was significant (F_(1,25)_ = 5.277, p = 0.03 η^2^ = 0.174; see Fig. 4). Post-hoc comparisons showed a significant improvement in the accuracy of the AVG group (pre-training mean = 11.28; SE = 0.97; post-training mean = 15.12; SE = 0.8, p = 0.002, Cohen’s d = 1.09), whereas the NAVG group did not show any significant improvement (pre-training mean = 11.13; SE = 1.12; post-training mean = 10.85; SE = 0.93). Comparing the mean changes between AVG and NAVG groups, Cohen’s d was = 0.9.

### Focused visuo-spatial attention

The accuracy (rate of the number of correct items) of non-verbal symbols identification in the focused visuo-spatial attention task was analyzed to measure the effect of the two different trainings on the visuo-perceptual information processing.

Accuracy was analyzed using a 2 (time: pre vs. post) X 2 (intervention group: AVG vs. NAVG) ANCOVA. To exclude the possible effect of educational experience, chronological age was controlled by entering it as covariate.

**Figure 5:**
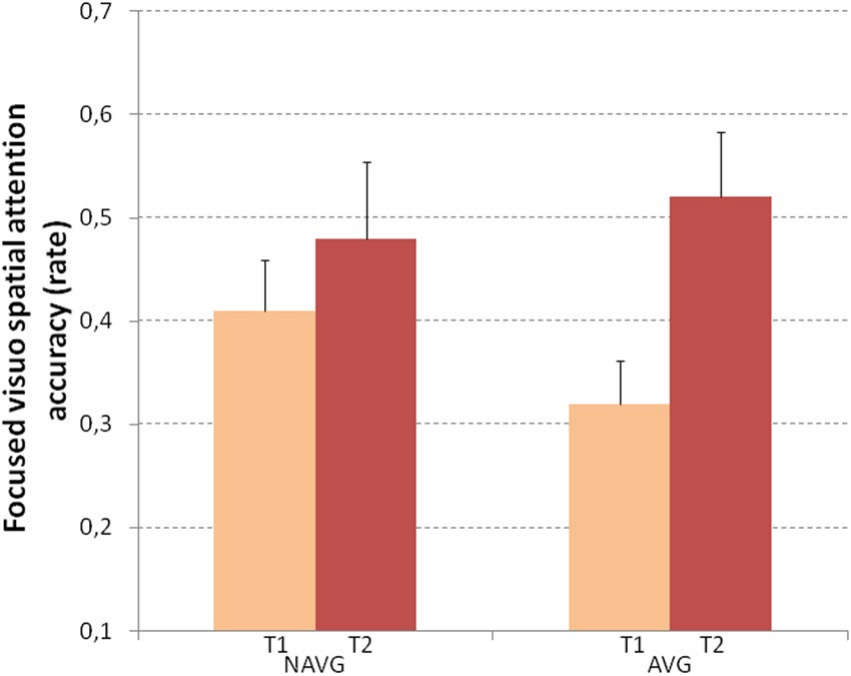
Focused visuo-spatial attention was measured before (T1) and after (T2) NAVG and AVG training in English-speaking children with dyslexia. Significant improvement in focused visuo-spatial attention was observed only after AVG training. Error bars represent standard errors.

The time X group interaction was significant (F_(1,25)_ = 4.687, p = 0.04 η^2^ = 0.158; see Fig. 5). Post-hoc comparisons showed a significant improvement in the visuo-spatial attention accuracy of the AVG group (pre-training mean = 0.32; SE = 0.042; post-training mean = 0.52; SE = 0.063, p = 0.001, Cohen’s d = 0.95), whereas the NAVG group did not show any significant improvement (pre-training mean = 0.41; SE = 0.049; post-training mean = 0.48; SE = 0.074). Comparing the mean changes of AVG and NAVG groups, Cohen’s d was = 0.85.

### Distributed visuo-spatial attention

The accuracy (rate of the number of correct items) of non-verbal symbols identification in the distributed visuo-spatial attention task was assessed in order to measure the effect of the two different trainings on the visuo-perceptual information processing.

Accuracy was analyzed using a 2 (time: pre vs. post) X 2 (intervention group: AVG vs. NAVG) ANCOVA. To exclude the possible effect of educational experience, chronological age was controlled by entering it as covariate. No main effect or interaction was significant.

### Visual, auditory and audio-visual processing

The inefficiency index (speed to accuracy ratio, i.e., msec./accuracy rate) in the localization of auditory, visual and audio-visual stimuli, was analyzed to measure the effect of the two different trainings on the unisensory and multisensory processing.

The inefficiency index was analyzed using two mixed ANCOVAs with 2 (time: pre vs. post) X 2 (intervention group: AVG and NAVG) design.

In both the unisensory and multisensory ANCOVAs no main effect or interaction were significant (see Supplementary Information for details).

### Cross-sensory attentional shifting

**Figure 6:**
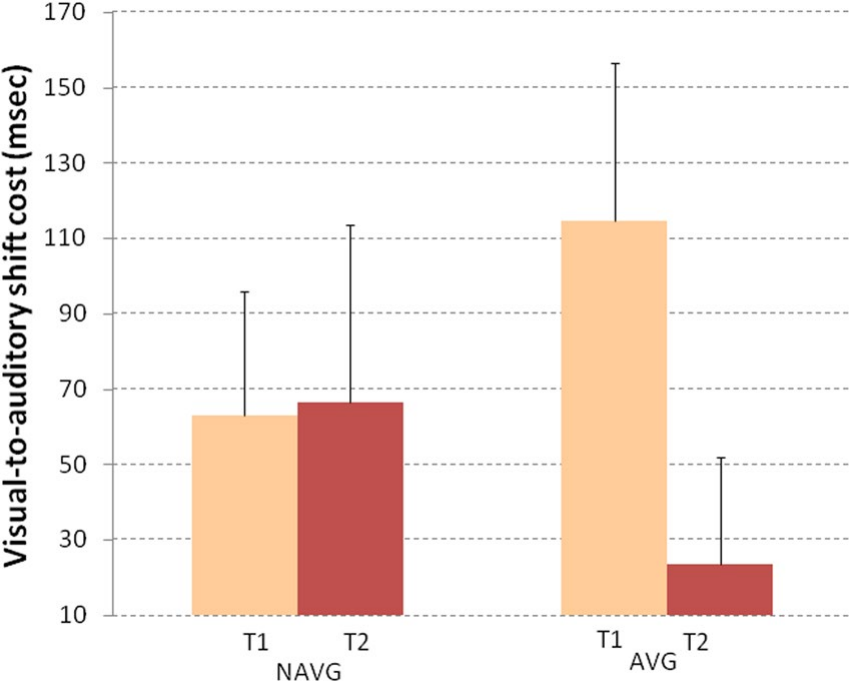
Visual to auditory shift costs (in msec.) were measured before (T1) and after (T2) NAVG and AVG trainings in English-speaking children with dyslexia. Visual to auditory shift cost significantly decreased only after AVG training. Error bars represent standard errors.

Similarly to Harrar *et al*.’ s analysis^44^, we calculated unisensory accurate reaction times (RTs) for each child both, when the previous stimulus was the same (e.g., two successive visual trials) and when it was different (e.g., a visual trial followed by an auditory trial). Visual (i.e. from auditory to visual) and auditory (i.e. from visual to auditory) shift costs were calculated by computing the difference between RTs on consecutive trials with the same target, and RTs when the previous trial was different. Cross-sensory shift costs were analyzed using a mixed 2 (time: pre vs. post) X 2 (target modalities: visual-to-auditory vs. auditory-to-visual) X 2 (intervention group: AVG and NAVG) ANCOVA. To exclude that differences in educational experience and pre-training cross-sensory attentional shifting abilities could drive the observed results, chronological age as well as attentional shift performance in T1 were controlled by entering them as covariates. The three-way time X target modality X intervention group interaction was significant when the cross-sensory attentional shifting was measured as shift costs (F_(1,23)_ = 8.923, p = 0.007, η^2^ = 0.280). For each group a mixed ANOVA with a 2 times (time: pre vs. post) X 2 (target modalities: visual-to-auditory vs. auditory-to-visual) design was performed. The time X target modality interaction was significant only in the AVG group (F_(1,15)_ = 4.782, p = 0.045, η^2^ = 0.242). Within-subject planned comparisons showed that only the visual-to-auditory shift cost was significantly reduced after the AVG training (pre-training mean = 115 msec., SE = 41.90 and post-training mean = 24 msec., SE = 28.57; t_(15) _= 1.765, p = 0.049, Cohen’s d = 0.65; see Fig. 6), indicating that AVG training improved the attentional shifting from visual to auditory modality. Comparing the mean changes between AVG and NAVG groups, Cohen’s d was = 0.47.

### The link between reading speed and cognitive improvements

We performed a two-step, fixed entry, multiple regression analysis on the entire sample of children with dyslexia (n = 28). The dependent variable was the general reading speed changes (i.e., the mean between the word and pseudoword reading tasks changes in seconds between T1 and T2), and the predictors were: (1) age; (2) phonological working memory, visuo-spatial attention and attentional shifting changes. Phonological working memory changes were indexed by calculating the difference between T2 and T1 performance in the auditory-phonological working memory tasks. Visuo-spatial attention changes were indexed by calculating the difference between the improvements (T2 − T1 rate accuracy) in focused vs. distributed condition. Cross-sensory attentional shifting changes were indexed by calculating the difference between the cost reduction (T1 − T2 costs) in the visual-to-auditory vs. auditory-to-visual condition.

Phonological, visuo-spatial and cross-sensory attentional enhancements accounted for 33% of the unique variance of reading speed improvements (p = 0.025), demonstrating that both working memory and attentional functioning improvements were involved in the reading remediation in children with dyslexia.

To measure the unique effect of attentional improvements on reading speed improvements - controlling for training-induced phonological working memory changes - we performed a three-step, fixed entry, multiple regression analysis in which the predictors order were: (1) age (2), phonological working memory and (3) attention changes. Only the phonological improvements significantly accounted for a significant quote of variance of reading speed changes (18%, p = 0.028). Conversely, to measure the unique effect of phonological working memory improvements on reading speed changes - controlling for training-induced attentional changes - we performed a three-step, fixed entry, multiple regression analysis in which the predictors order were: (1) age (2), attentional and (3) phonological working memory changes. Only the attentional improvements significantly accounted for a quote of variance (25%, p = 0.032), indicating that working memory and attentional predictors share a quote of variance for explaining the reading speed improvement.

### Video games improvements

To measure the improvement in the video game abilities of the two training types, both groups were evaluated before every training session on a single mini-game (“Bunnies Don’t Like Being Disturbed on Holiday” for the AVG and “Bunnies Don’t Give Gifts” for the NAVG training). The z-scores from the video game scores were calculated. A 2 (time: pre vs. post training) X 2 (intervention group: AVG vs. NAVG) mixed ANOVA was performed. The time main effect was significant (F_(8,208)_ = 13.18, p < 0.0001, η^2^ = 0.34), showing overall improvement in game performance. Importantly, the time X group interaction was not significant, indicating that the two groups of children with dyslexia similarly improved in terms of their video game abilities during the two trainings. Within-subjects planned comparisons (paired sample t-tests) revealed a significant improvement (i.e., mean z-score for day one vs. day nine) in both AVG (mean = −0.07 SE = 0.26 vs. mean = +1.45 SE = 0.17; t_(15)_ = −5.9, p < 0.0001) and NAVG players (mean = −0.12 SE = 0.30 vs mean = +1.51 SE = 0.39; t_(11)_ = −3.22, p = 0.008).

## Discussion

Most of the world’s writing systems use the one-letter-one-phoneme mapping principle that also characterizes the Italian orthography. We previously demonstrated an improvement in word reading and phonological decoding efficiency after AVG training in a sample of Italian children with dyslexia^20^. The reading efficiency improvements after the AVG training were characterized by increased reading speed without any cost in accuracy. Similar findings on reading speed were replicated in another sample of Italian children with dyslexia in which children were treated with both, NAVG and AVG trainings (26, Experiment 3). These reading speed improvements are relevant, because the single most salient and universal fact about skilled reading is the remarkable speed and apparent effortlessness word identification^54^.

However, the reading speed improvements obtained in Italian, a shallow orthography, were criticized on the basis of the high degree of grapheme-to-phoneme consistency of the language, which does not afford many alternative pronunciations^50^. In contrast, English orthography is complex and involves multiple-sized units, therefore the rules of grapheme-to-phoneme mapping need more time to be acquired. English-speaking children with dyslexia need more exposition to the complex grapheme-to-phoneme mapping rules to obtain better accuracy^8^. Our non-conventional remediation program targets a faster and better extraction of the already acquired grapheme-to-phoneme mapping rules, without any cost in accuracy. Accuracy is not expected to change, as AVG training does not involve any grapheme-to-phoneme mapping learning.

In our study with a sample of English-speaking children with dyslexia, we observed an improvement in word reading and phonological decoding speed, without any cost in accuracy. These findings demonstrate that, even in a language with deep orthography, AVG training improves reading skills without a direct targeting of phonological, orthographic or grapheme-to-phoneme decoding. These results are in line with the improved speed of processing and reading speed already found with AVG^55^, also in patients with amblyopia^56^. Thus, in children with dyslexia, AVG training enables an enhancement of processing speed and reading.

One explanation of the improvements driven by the use of AVG is linked to the domain-general training characterized by the complexity of AVGs^48^. In visual search and in letter recognition with flankers, it has been demonstrated that AVG training ameliorates the accumulation of information rate over time and consequently the efficiency of the decision-making process^57–61^. In our experiment, children with dyslexia may have improved their abilities to extract the relevant information^33, 34^, accelerating letter string decoding. This interpretation is supported by the results in our focused condition of the visuo-spatial attention task in which attention is cued in a specific location. The attentional processing improvement was present only after AVG training. In contrast to the previous AVG training study in Italian children with dyslexia^20^, in the present report, we found no improvements in distributed visuo-spatial attention. It is difficult to hypothesize whether these different training effects could be due to differences in orthographies of the two samples. The learning of different orthographies could involve and/or train different attentional processing (e.g., smaller or larger attentional focus), and consequently induce different treatment sensibility. However, considering that most of the AVG training studies^45, 47, 48, 50, 52, 53, 55, 57–61^ involved English-speaking participants, this hypothesis appears quite difficult to be sustained, unless we hypothesize that English-speaking children with dyslexia have a specific deficit in distributed attention that can hardly be changed. Even if this hypothesis was true, the present study shows that an improvement in focused attention is however sufficient in order to improve reading abilities.

Because of the unexpected non significant effects of the AVG in comparison to the NAVG training on distributed attention, we measured the effect of the attentional improvements derived by the use of AVG observed in the focused condition, controlling the possible noise effect related to the use of other kind of videogames observed in the distributed condition^62^. This finding suggests that rapid orienting of visual attention plays an important role in reading remediation also in deep orthographies characterized by complex multiple-sized units. Accordingly, the efficient orienting of attention at pre-reading stage was a good predictor of future reading skills in different orthographies^14, 16^.

Our unconventional training also increased phonological short-term memory and phoneme blending skills. Consequently, playing AVG may also improve the phonological working memory deficit usually associated with dyslexia^5^. Indeed, no phonological information was presented during the training, therefore no direct training of phonological working memory was carried out. Originally, Green and Bavelier^47^ demonstrated that AVG can improve a range of spatial and temporal aspects of visual attention, not strictly connected to the ones directly trained by the video game use per se. The authors also showed that a better use of sensory evidence (or target filtering) could be obtained by AVG players in tasks that involved not only visual, but also auditory stimuli^48^. These findings demonstrated that a visual attentional training could produce a general beneficial effect also on cognitive functions that were not directly trained by AVG, such as the auditory processing, and, consequently, phonological short term memory. By using a complex verbal span task that required both mental calculation and short term memory, Oei and Patterson^62^ showed a specific effect of AVG on phonological working memory. These data were confirmed by our 12-hour training. Similar findings have recently been highlighted by Lawton^63^, who showed how a motion discrimination training could improve phonological working memory and reading skills in English-speaking children with dyslexia.

Harrar *et al*.^44^ interpreted the multisensory integration deficit as a tendency to extend the time spent on visual stimuli when attention has to be shifted from visual to auditory stimuli. Our findings in the cross-sensory attentional shifting analysis demonstrate, for the first time, that English-speaking children with dyslexia trained with AVG specifically improve their cross-sensory attentional shifting ability from visual to auditory stimuli. This result could be linked to an increased connection between occipito-temporal orthographic and temporo-parietal phonological areas^43^.

Since video-games scores improved similarly in both video game trainings, the different effects in reading, memory, visuo-spatial and cross-sensory attentional skills can not be due to differences in the games training engagement.

Finally, the first regression analysis indicates that reading improvements are connected both with attentional and working memory gains, because visuo-spatial, cross-sensory attentional and phonological working memory changes explain more than 30% of variance in reading speed acceleration. The second and third regression analysis show that both attentional processing and phonological working memory play a significant and partially overlapping role in reading speed improvement. Indeed, recent evidence from neurophysiological studies demonstrated that top-down modulation serves as a common neural mechanism underlying working memory as well as cross-sensory attention^64, 65^. Spatial cues - directing attention to external stimuli or to content in working memory - activate a network spanning from frontal eye fields, presupplementary motor cortex and anterior cingulate cortex, to the intraparietal sulcus, and the superior parietal lobule. This overlap has been confirmed in multiple neuroimaging studies and a meta-analysis^66^.

In conclusion, our results demonstrate the possible causal role, for dyslexia, of working memory, visuo-spatial and visual-to-auditory attentional shifting. These findings pave the way for low-resource-demanding remediation programs that could reduce the severity of reading disorders in children who read an orthographically deep language.

## Materials and Methods

### Participants

Twenty-eight dyslexic children (8 females and 20 males), mean age 10.1 years (range 7.8–14.3) were involved in the experiment. All the children were recruited through school newsletters or dyslexia associations. The participants’ parents completed an interview and a videogame questionnaire tagging their children’s gaming habits: these included game types and time spent video gaming each week.

The children were recruited according to four criteria, all confirmed by their parents upon signing the study’s consent form: (i) confirmed diagnosis of dyslexia, (ii) no history of psychiatric or neurological disease, (iii) no exposure to AVG in the last six months, and (iv) commitment not to play videogames at home in the course of the study.

In addition, in order to be included in the study, children needed to have an average IQ, normal or corrected to normal visual acuity, no ADHD diagnosis. Children were randomly allocated to either AVG (n = 16) or NAVG training (n = 12, see Table 2 for details).

**Table 2:** Summary behavioral characterization of participants. ^a^ = ^67^.

	AVG Group (n = 16)	NAVG Group (n = 12)	t-value (p)
Age (years)	9.8 (1.4)	10.9 (1.9)	−1.682_(26)_ (0.104)
TOWRE “Sight words”^a^ (z-score)	−1.6 (0.76)	−1.77 (0.42)	0.645_(26)_ (0.525)
TOWRE “Phonemic Awareness”^a^ (z-score)	−1.13 (0.75)	−1.42 (0.69)	1.034_(26)_ (0.311)
Phonological short-term-memory (correct item)	9.44 (6.12)	11.25 (4.63)	−1.035_(26)_ (0.310)
Phoneme blending (correct item)	12.3 (3.8)	12.08 (3.48)	0.163_(26)_ (0.871)
Non Action Video Game Experience (hours per week)	1.56 (2.03)	2.11 (2.59)	0.635_(26)_ (0.531)

### Apparatus, Stimuli and Procedure

#### Training procedure

The training procedure was the same used in Franceschini *et al*.^20^ and Gori *et al*.^26^: participants were tested 3 to 5 days before starting the treatment and re-tested between one and three days after its end. Video games were played at about 150 cm from a 23-in Dell Optiplex 9030 ﻿VAIO﻿﻿ Screen. A commercial Wii™ video game from Ubisoft™ (deemed suitable for children age 7 and older by the Pan European Game Information) called “Rayman Raving Rabbids” was used. Single mini-games were selected from the overall game and categorized as AVG or NAVG. In order to classify the mini-games, the checklist developed by Green *et al*.^48^, was followed: all AVGs share a set of qualitative features, including (1) extraordinary speed both in terms of very transient events and in terms of the velocity of moving objects; (2) a high degree of perceptual, cognitive, and motor load in the service of an accurate motor plan; (3) unpredictability both temporal and spatial; (4) an emphasis on peripheral processing. We labeled AVGs only the mini-games that presented all the four characteristics listed above, whereas NAVGs presented not more than one of them. NAVG participants did not see the mini games used by the AVG player and viceversa. Each child was individually treated for 9 sessions of 80 minutes a day during two weeks.

#### Tasks administration and evaluation

All reading performance of participants were recorded and time and errors were coded by native-english speakers. The experimenters were blind regarding the participants’ allocation to AVG or NAVG group.

#### Reading task

Word reading: The Sight Words task, form “A” of Towre 2^67^ was used in T1, form “B” in T2. In both cases, participants were asked to read the first three columns (81 words; “long” lists) as fast and accurately as possible. In addition, the first column of form “C” (including 27 words “short” list) was used in T1 and the first column of form “D” (including 27 words; “short” list) was used in T2. Again, participants were asked to read as fast and accurately as possible. We selected different reading tests in T1 and T2 evaluations to exclude the test-retest effect. Time (in sec.) and numbers of errors were recorded. Performance in the two lists were mediated for the statistical analysis. One error was assigned if the word was not pronounced entirely correctly. Self corrections were not considered errors. The tasks were administered in about 10 minutes.

Phonological decoding: The Phonemic Awareness task, form “A” of Towre 2^67^ was used in T1, form “B” in T2. In both cases, participants were asked to read the first two columns (44 pseudowords; “long” lists) as fast and accurately as possible. In addition, the first column of form “C” (including 22 pseudowords; “short” list) was used in T1 and the first column of form “D” (including 22 pseudowords; “short” list) was used in T2. Again, participants were asked to read as fast and accurately as possible. We selected different reading tests in T1 and T2 evaluations to exclude the test-retest effect. Time (in sec.) and numbers of errors were recorded Performance in the two lists were mediated for the statistical analysis. One error was assigned if the pseudoword was not pronounced entirely correctly. Self corrections were not considered errors. The tasks were administered in about 10 minutes.

#### Auditory-phonological working memory

Children listened to a series of pseudoword trigrams using professional headphones. Children had to repeat each trigram in the correct sequence. Two lists of trigrams were presented. If the children repeated correctly at least one of them, a new series with an additional couple of trigram lists was proposed. If both lists were wrongly reported, the task was interrupted. One point for each correctly repeated item was assigned. The series started with two trigrams and continued up to a maximum of eight trigrams.

Phoneme blending: Two lists of words (10 + 10) were presented. The first list differed in T1 and T2 (the same sound but in reversed order was presented in T1 and T2: T1 “day” and T2 “aid”; T1 “tar” and T2 “art”), the second list was the same. The two lists were counterbalanced among subjects. The instructions for children were: “your task is to put some sounds together to create a word. If I pronounced the sounds /D/-/A/-/D/ what word would be created? Try to blend those sounds together to figure out the word”.

The sounds were recorded by an Australian native speaker and the children were required to put together the sounds (delivered to them by means of professional headphones) in order to figure out a word. One point was assigned if the word was recognized, zero points if the word was not recognized. The tasks were administered in about 10 minutes.

#### Focused and distributed visuo-spatial attention

The experimental procedure and data acquisition were controlled using E-prime 2.0 (Psychology Software, Inc.) running on a 23-in Dell Optiplex 9030 VAIO Screen. The viewing distance was set to 50 cm, with the vertical body midline aligned with the screen center by using the chinrest. The fixation mark was a green square (0.3° × 0.3°). A string of six black, non-verbal symbols (1.1° × 1.8°), three for each half of the visual field (eccentricity 1.1°, 3.6° and 6.1°), were displayed simultaneously. The target was the non-verbal symbol indicated by a red dot (0.3°) that appeared before (focused attention condition) or after (distributed attention condition) the string, and a post-mask (six 8-like red figures string, 1.1° × 1.8°) was displayed after six black, non-verbal symbols. All the stimuli were presented on a white background and had a luminance of 24 cd/m^2^. The two experimental sessions (i.e. focused and distributed) were mixed.

Participants were instructed to keep their eyes on the fixation point for all the duration of the trial. Each trial started with the display of the fixation point for 1000 msec. In the focused condition, a red dot cued attention on the target location, appearing for 34 msec. before the string of six black non-verbal symbols, which appeared for 150 mses. In the distributed condition, the red dot appeared immediately after the six symbols disappearance. Then, a blank screen for 100 msec. was presented. A post-mask was displayed for 50 mses., and a blank screen for 1000 msec. Participants were instructed to identify the target within eight possible alternatives (i.e., chance level = 0.125), without time limit. Responses were pointed by the participant and entered by the experimenter who pressed the corresponding key on the computer keyboard. No feedback was provided. The experimental session consisted of 96 trials.

The tasks were administered in about 15 minutes.

#### Visual, auditory, audio-visual processing and cross-sensory attentional shifting

The experimental procedure and data acquisition were controlled with E-prime 2.0 (Psychology Software, Inc.) running on a 23-in Dell Optiplex 9030 VAIO Screen. The viewing distance was set to 50 cm, with the vertical body midline aligned with the screen center by using the chinrest. The visual target stimulus was a black square (2.5 × 2.5°) presented on a light grey background at an eccentricity of 16° from the fixation point (0.5 × 0.5°). The sound target stimulus was a 500-Hz sound (pure tone) and was presented in one of the 2 (left or right) external speakers. Speakers were positioned close to the left and right screen borders, and were elevated so that the center of the speakers was aligned with the monitor horizontal median line, where the visual stimulus was presented. This way we ensured that visual and auditory stimuli were presented close together in space. On each trial, the fixation point appeared for a random duration between 1000 and 2500 msec., in order to avoid the possibility that participants might build a prediction about the target onset time over the course of the trials. Subsequently, the target stimulus appeared according to the 3 possible experimental conditions. In the “visual” condition, the visual stimulus was presented alone for 200 msec. in the left or right visual hemifield. In the “auditory”, the sound was presented alone for 200 msec. in the left or right speaker. In the “audio-visual” condition, a synchronized combination of the visual and auditory stimulus was presented for 200 msec., always on the same side (left visual hemifield/left speaker or right visual hemifield/right speaker). Participants were asked to respond as fast and as accurately as possible by pressing the letter “Z” for any stimulus appearing on their left side and the letter “M” for any stimulus appearing on their right side. The maximum time for response was set to 2000 msec. The experimenter controlled the transition from one trial to the next. After 10 practice trials, participants performed 90 experimental trials (3 conditions × 2 sides × 15 repetitions) and 10 catch trials (where no visual or auditory stimulus occurred), randomly intermixed, for a total duration of approximately 15 min.

Informed written consent was obtained from the parents of each child; the University of Sydney ethic committee approved the research protocol (p. n. 2015/059). The entire research process was conducted according to the principles expressed in the Declaration of Helsinki.

## Electronic supplementary material

Supplementary information
