# Supplementary material for: Action video games improve reading abilities and visual-to-auditory attentional shifting in English-speaking children with dyslexia
Source: Sci Rep. 2017 Jul 19;7:5863. doi: 10.1038/s41598-017-05826-8 (PMC5517521; doi:10.1038/s41598-017-05826-8)
Supplement: Supplementary file 1 — Supplementary information [file 41598_2017_5826_MOESM1_ESM.doc]

**Action video games improve reading abilities and visual-to-auditory attentional shifting in English-speaking children with dyslexia**

Sandro Franceschini^1,2§^*, Piergiorgio Trevisan^3§^, Luca Ronconi^1,2,4^, Sara Bertoni^1^,

Susan Colmar^5^, Kit Double^5^, Andrea Facoetti^1,2^ & Simone Gori^2,6^

1. Developmental and Cognitive Neuroscience Lab, Department of General Psychology, University of Padua, Padova 35131, Italy.
2. Child Psychopathology Unit, Scientific Institute "E. Medea", Bosisio Parini, Lecco 23842, Italy.
3. Department of Languages and Literatures, Communication, Education and Society, University of Udine, Udine 33100, Italy.
4. Center for Mind/Brain Sciences, University of Trento, Rovereto, Trento 38068, Italy.
5. Faculty of Education and Social Work, University of Sydney, NSW 2006, Australia.
6. Department of Human and Social Sciences, University of Bergamo, Bergamo 24129, Italy.

§S.F. and P.T. equally contributed to this work

**SUPPLEMENTARY INFORMATION**

**Supplementary results**

**Visual, auditory and audio-visual processing**

The planned comparisons with age as a covariate for both Unisensory and Multisensory modalities indicate that only AVG training can improve both Unisensory and Multisensory processing (AVG mean T1= 762.20, SE= 60.16; mean T2= 597.93, SE= 24.64, p=.004; and mean T1= 511.63, SE= 31.07; mean T2= 448.232, SE= 24.85, p=.011; vs. NAVG mean T1= 683.80, SE= 69.98; mean T2= 592.55, SE= 28.67, p=.148; and mean T1= 474.20, SE= 36.14; mean T2= 472.02, SE= 28.90, p=.936; respectively).

**Supplementary methods**

**Phoneme blending**

Materials

|  | **T1** | **T2** |
| --- | --- | --- |
| 1 | Sigh | Ice |
| 2 | Ape | Pay |
| 3 | Toe | Oat |
| 4 | Eat | Tea |
| 5 | Jay | Age |
| 6 | Aid | Day |
| 7 | Lie | Aisle |
| 8 | Art | Tar |
| 9 | car | Ark |
| 10 | aim | May |
| **11** | *Winds* | *Winds* |
| **12** | *block* | *block* |
| **13** | *Drive* | *Drive* |
| **14** | *Swung* | *Swung* |
| **15** | *Chest* | *Chest* |
| **16** | *picked* | *picked* |
| **17** | *Tracks* | *Tracks* |
| **18** | *breathe* | *breathe* |
| **19** | *Robot* | *Robot* |
| **20** | *plat* | *plat* |
